# Clinical experience of MRI^4D^ QUASAR motion phantom for latency measurements in 0.35T MR‐LINAC

**DOI:** 10.1002/acm2.13118

**Published:** 2020-12-18

**Authors:** Taeho Kim, Benjamin Lewis, Rajiv Lotey, Enzo Barberi, Olga Green

**Affiliations:** ^1^ Department of Radiation Oncology Washington University School of Medicine St Louis MO USA; ^2^ ViewRay Inc Oakwood Village OH USA; ^3^ ModusQA London ON Canada

**Keywords:** latency, MR‐LINAC

## Abstract

**Purpose:**

In MRgRT, accuracy of treatment depends on the gating latency, when real‐time targeting and gating is enabled. Gating latency is dependent on image acquisition, processing time, accuracy, efficacy of target tracking algorithms, and radiation beam delivery latency. In this report, clinical experience of the MRI^4D^ QUASAR motion phantom for latency measurements on a 0.35‐T magnetic resonance‐linear accelerator (MR‐LINAC) with two imaging speeds and four tracking algorithms was studied.

**Materials/Methods:**

Beam‐control latency was measured on a 0.35‐T MR‐LINAC system with four target tracking algorithms and two real‐time cine imaging sequences [four and eight frames per second (FPS)]. Using an MR‐compatible motion phantom, the delays between phantom beam triggering signal and linac radiation beam control signal were evaluated for three motion periods with a rigid target. The gating point was set to be 8 mm above the full exhalation position. The beam‐off latency was measured for a total of 24 combinations of tracking algorithm, imaging FPS, and motion periods. The corresponding gating target margins were determined using the target motion speed multiplied by the beam‐off latency.

**Results:**

The largest measured beam‐off latency was 302 ± 20 ms with the Large Deforming Targets (LDT) algorithm and 4 s motion period imaged with 8‐FPS cine MRI. The corresponding gating uncertainty based on target motion speed was 3.0 mm. The range of the average beam‐off latency was 128–243 ms in 4‐FPS imaging and 47–302 ms in 8‐FPS imaging.

**Conclusions:**

The gating latency was measured using an MRI^4D^ QUASAR motion phantom in a 0.35‐T MR‐LINAC. The latency measurements include time delay related to MR imaging method, target tracking algorithm and system delay. The gating uncertainty was estimated based on the beam‐off latency measurements and the target motion.

## 1 INTRODUCTION

Tumors in the thorax and abdomen can move significantly, travelling up to 5 cm during respiration.^1^, ^2^ In addition to a large motion range, tumor motion is not consistent from one breathing cycle to another.^3^, ^4^ Magnetic Resonance‐guided radiotherapy (MRgRT) is a highly desirable image‐guided radiotherapy (IGRT) modality for moving targets because it provides continuous imaging of patient anatomy with the superior soft tissue contrast provided by MRI, without the ionizing radiation imparted by x‐ray imaging, during radiation beam delivery. Currently, MRgRT with real‐time two‐dimensional (2D) tumor tracking is clinically available^5^, ^6^ and MRgRT treatments have been demonstrated to produce excellent local control rates with little toxicity.^5^, ^7^, ^8^ Tumors benefiting most from real‐time 2D tumor tracking are those near the diaphragm where organs experience the maximal respiratory motion, including the lower lobe of the lungs and the liver dome.

Magnetic Resonance‐guided radiotherapy systems with simultaneous 2D tumor tracking capability consist of an MRI system for anatomical imaging and a radiotherapy machine for radiation beam delivery in real‐time. Accuracy of treatment when real‐time targeting and gating is enabled depends on the latency of gating processes including imaging, target tracking, and radiotherapy machine control.^2^, ^6^, ^9^ The gating latency can be affected by the speed of image acquisition and processing, accuracy and efficacy of target tracking algorithms, and radiation beam delivery latency. Any substantial latency can induce a systematic deviation of the delivered dose from the planning dose. A general recommendation for gating latency comes from AAPM TG‐142, which suggests that the temporal accuracy of phase or amplitude‐based gating should be within 100 ms of the baseline latency.^10^ Previously, gating latency has been measured using a moving phantom and an independent dosimetric device such as film or diode detectors.^11^, ^12^ When detecting the moving target in a gating window (gating boundary for 2D motion), a monitoring system sent a beam‐control signal to a radiation delivery system. Delay from the time of the initiating beam‐control signal by the monitoring system to the time of the dosimetric measurement was considered as the gating latency.^12^ Also, a model of a delayed dosimetric measurement compared to a static dosimetic measurement on a film can be used to estimate the gating latency.^11^ This study demonstrates that dosimetric device‐based approaches can be readily applied to any delivery system but remain sensitive to temporal and dosimetric response of dosimetric devices.^11^

Instead of using dosimetry devices, the gating latency can be measured by obtaining a beam‐control signal directly from the radiation delivery system.^6^, ^13^ Delay from the time of the initiating beam‐control signal produced by the monitoring (or tracking) system to the time of radiation beam‐control signal provides the gating latency. This approach provided high temporal fidelity and consistent results but required a direct signal reading from the radiation delivery system.

Green et al. reported the gating latency of a 0.35‐T MRgRT system (MR‐^60^Co) using the direct signal reading approach.^6^ They used an MRI‐compatible programmable CIRS motion phantom for the gating latency measurements. The motion phantom had a moving insert for MR imaging, target tracking, and beam triggering. Once the insert was out of the gating boundary and the beam‐off signal was initiated by the gating system, a rod connected to the insert of the phantom triggered physical sensors which sent an electrical signal to an oscilloscope. The beam‐off signal from the radiation delivery system was imported to the oscilloscope and the time difference between the gating electrical signal and the beam‐off signal was reported as the beam‐off gating latency. This study produced an average latency of 394 ms with a range of 246 to 527 ms. This approach still requires software and equipment in addition to an MRI compatible motion phantom but removes uncertainty in latency measurements due to dosimetric devices.

Recently the MRI^4D^ QUASAR motion phantom (ModusQA, Ontario, Canada) was introduced for gating system tests including gating latency measurements. It has an interface to collect the beam‐control signal from the radiation delivery system and the gating signal from the motion phantom without an oscilloscope. In our institution, the gating latency measurements of a 0.35‐T magnetic resonance‐linear accelerator (MR‐LINAC) have been completed using an MRI^4D^ QUASAR motion phantom after the MRgRT system was upgraded with four tracking algorithms and two imaging speeds (four and eight frames per second (FPS)). In this report, we discuss our clinical experience of using the MRI^4D^ QUASAR motion phantom for latency measurements on a 0.35‐T MR‐LINAC. The following items are briefly discussed: (a) the new features of the imaging techniques and tracking algorithms of 0.35‐T MR‐LINAC, (b) the clinical experience of the gating latency measurements with two imaging speeds and four tracking algorithms, and (c) gating geometric uncertainty according to the measured gating latency.

## 2 MATERIALS AND METHODS

The gating latency depends on the speed of image acquisition and processing, accuracy and efficacy of target tracking algorithms, and radiation beam triggering latency. In the gating latency measurements, combinations of imaging and target tracking conditions were parameterized.

### 2.A 0.35‐T MR‐guided radiotherapy

A 0.35‐T MRgRT system (ViewRay Inc., Oakwood Village, Ohio) was used for the gating latency measurements. The MRgRT system consists of a 0.35‐T split‐doughnut superconducting MRI for real‐time imaging and a 6‐MV flattening‐filter‐free (FFF) LINAC for radiation delivery.^14^ Imaging for MRgRT included volumetric MRIs acquired using a steady‐state precession (TrueFISP) pulse sequence in an axial orientation for localizing treatment targets, and 2D TrueFISP cine MRIs acquired in a sagittal plane for target tracking.^6^ Since target tracking and radiation beam gating utilize real‐time imaging information, 2D cine MRI protocols were used in the gating latency measurements.

### 2.B Cine MRI with 4 and 8 FPS

Along with the system upgrade, an 8 FPS‐2D cine MRI protocol had been released to the clinic in addition to the previously available 4 FPS‐2D cine MRI protocol in a sagittal plane for use during MRgRT. The 4 FPS‐2D cine MRI protocol produces a set acquisition spatial resolution: 3.5 × 3.5 mm^2^ with 5, 7, or 10 mm slice thicknesses, using a Cartesian acquisition trajectory. Imaging parameters were TR/TE = 2.1/0.91 ms, flip angle = 60°, rBW = 1351 Hz/pixel, FOV = 350 × 350 mm^2^, and imaging matrix = 100 × 100. The 8 FPS‐2D cine MRI protocol produces an acquired spatial resolution of 2.4 × 2.4 mm^2^ with 5, 7, or 10 mm slice thicknesses, using radial acquisition trajectory. Imaging parameters were TR/TE = 500/1.38 ms, flip angle = 110°, rBW = 890 Hz/pixel, FOV = 350 × 350 mm^2^, imaging matrix = 144 × 144 and spokes: 176 (44 spokes updated per frame). In the gating latency measurements, the clinical 2D cine MRI protocols with 7 mm slice thickness were used.

### 2.C Target tracking algorithms

In addition to cine MRI with two imaging speeds, new tracking algorithms have been released to improve target tracking and gating.^15^ The tracking algorithms register images between a reference frame and a real‐time temporal frame. Since targets and organs‐at‐risk (OARs) have different degrees of structural deformation during MRgRT, suitable tracking algorithms are needed for particular applications. In addition to the previous tracking algorithm for general applications (called “Default”), three new tracking algorithms were released for specific applications. Table 1 includes the name and description of the tracking algorithms.

**Table 1:** Name and description of the tracking algorithms.

Name	Description
Default	General applications except stomach
Small mobile targets (SMT)	Applications for small targets with a large motion but a small deformation. Targets within liver (metastases), pancreas, kidneys, and near spine and heart
Large deforming targets (LDT)	Applications for large targets with a small motion but a large deformation. Whole organs such as prostate, bladder, liver, and lung
Complex mobile and deforming targets (CMDT)	Applications for large deforming targets with a large translational motion across the imaging plane. Stomach, GYN (cervix, uterus)

### 2.D Gating latency measurements

#### 2.D.1 Moving phantom and imaging insert

**Fig. 1:**
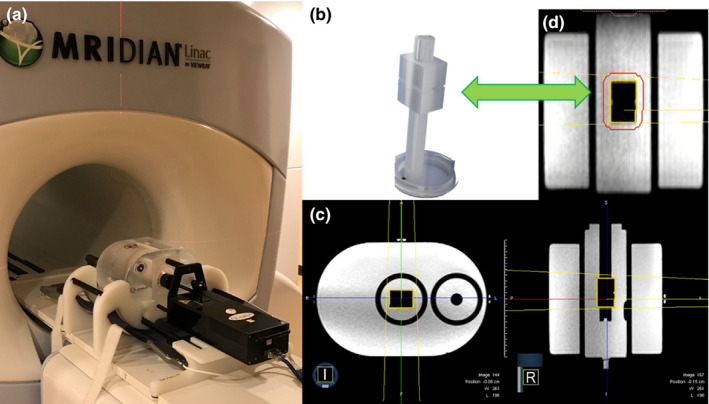
MRI^4D^ QUASAR motion phantom and imaging cuboid. (a) the phantom was installed on the phantom rack mounted on the couch top, (b) imaging cuboid attached to the stem, (c) cine MRI of the phantom in a sagittal plane, and (d) three‐dimensional magnetic resonance imaging of the phantom in an axial and sagittal planes. The yellow contour is the target cuboid and two yellow lines indicate the radiation beam at Gantry 0 degrees. The void structure is the cuboid in the insert filled with distilled water and MnCl_2_ · 4H_2_O at 7ppm.

The MRI^4D^ QUASAR motion phantom was positioned on the phantom rack mounted on the couch top as shown in Fig. 1(a). One torso coil array (six coil elements) was inserted into a channel in the rack underneath the phantom. Once the phantom was positioned with external lasers, the second torso coil array (six coil elements) covered the phantom before the phantom was sent to machine isocenter.

The body of the phantom had two cavities for acrylic inserts filled with distilled water and an added aqueous solution of MnCl_2_ · 4H_2_O at 7ppm (at 0.35T: T1~800 ms; T2~50 ms). One insert had an acrylic stem with a space for an ion chamber, inserted into the right cavity (a reference insert for dosimetric measurements). A dovetail collar was used to hold the reference insert in place. The other insert had an acrylic stem with a central cuboid target (3 × 4 × 5 cm^3^) for imaging and a space for an ion chamber (a moving insert for dosimetric measurements or imaging), inserted into the central cavity shown in Fig. 1(b). The insert was mounted to a drive rod. Since cine MRI was acquired in a sagittal plane, only the insert in the central cavity was visible on the MRI display screen in Fig. 1(c).

An electronic control box was placed in the radiofrequency (RF) cabinet room. Motor control cables were connected between the drive unit of the phantom in the MR room and the control box in the RF cabinet room through a waveguide with brass sponges to prevent RF interference. Using a CAT5e ethernet cable, a laptop was connected to the control box from the operating area. This allowed the gating signal from the motion phantom to be recorded and full control of the moving insert from the operating area. It is noted that beam‐control signal from the LINAC was sent from the control cabinet to the control box using a direct connection via coaxial cable (to Analog input2 port) which was not digitally filtered and had an inherent latency of <1 ms, which was verified by the authors from vendors.

#### 2.D.2 Beam gating setup and measurement

Volumetric MRI of the phantom (image resolution: 1.5 × 1.5 × 1.5 mm^3^) with the imaging insert at the end of exhalation position (static condition) was acquired using 3D TrueFISP MRI prior to the gating latency measurements shown in Fig. 1(d). A treatment plan was prepared using a fixed single beam at Gantry 0 degree on the cuboid target (Prescription dose: 100 Gy for uninterrupted beam delivery during the measurements).

**Fig. 2:**
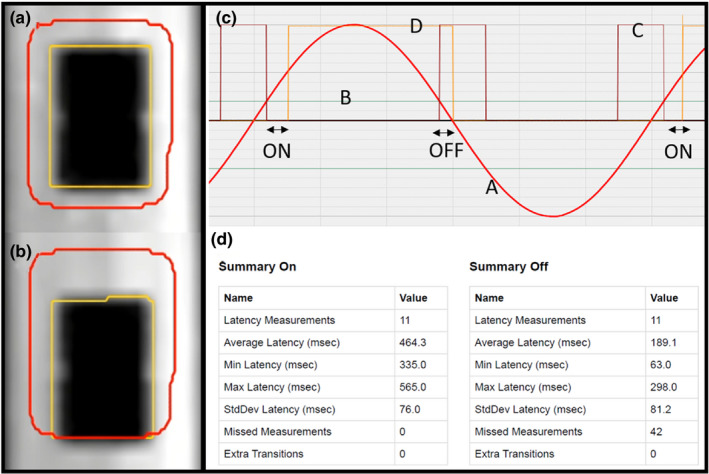
Gating setup. (a) target contour (yellow) and 8 mm margined gating boundary (red) at the end of exhalation position and (b) 8 mm shift inferior of the imaging insert on cine MRI. (c) 6 s motion period with 10 mm amplitude of the phantom motion control. (A: sine curve, B: gating position, C: triggering window, D: beam‐on signal from 0.35T MRgRT system) (d) latency measurement report of beam‐on and beam‐off generated by the phantom control software.

With the gating latency measurement setup, volumetric MRI of the phantom with the imaging insert at the end of exhalation position (static condition) was acquired to verify the target position. In the gating target setup, an 8‐mm margin was added to the target contour (yellow contour) as a gating boundary (red contour) shown in Fig. 2(a).

In the treatment delivery system (TDS) imaging window prior to the beam delivery screen, the imaging insert was shifted 8 mm inferior to touch the gating boundary using the phantom control software while cine MRI was acquired shown in Fig. 2(b). The shift initiated an estimate of the target‐out value as a percentage of the tracking contour outside of the gating boundary; a target‐out value of ~5% was acceptable. If the target‐out percentage was <5%, the imaging insert was shifted further in the inferior direction within a half‐step of the image resolution (1.75 mm). The shift of the imaging insert was used to set up the gating position in the phantom control software, and determined the position that would send the gating signal from the phantom control box to the control software shown in Fig. 2(c).

Once the gating position was determined in the phantom control software, the imaging insert was programmed for sinusoidal motion with three periods (4, 5, 6 s), 10 mm in amplitude (20 mm for peak‐to‐trough), during the gating latency measurements. Gating latency was determined as the delay from when the phantom control software reached the defined gating position and when the beam control signal was generated by the LINAC. Each measurement included at least 10 beam‐on and 10 beam‐off events. The beam‐off latency was measured for a total of 24 combinations: three motion periods, two imaging speeds, and four tracking algorithms.

#### 2.D.3 Gating uncertainty determination

The beam‐off latency was utilized to estimate a beam delivery uncertainty to the target. The corresponding gating uncertainty were determined using the target motion speed multiplied by the beam‐off latency.

## 3 RESULTS

### 3.A Gating Latency measurements

**Fig. 3:**
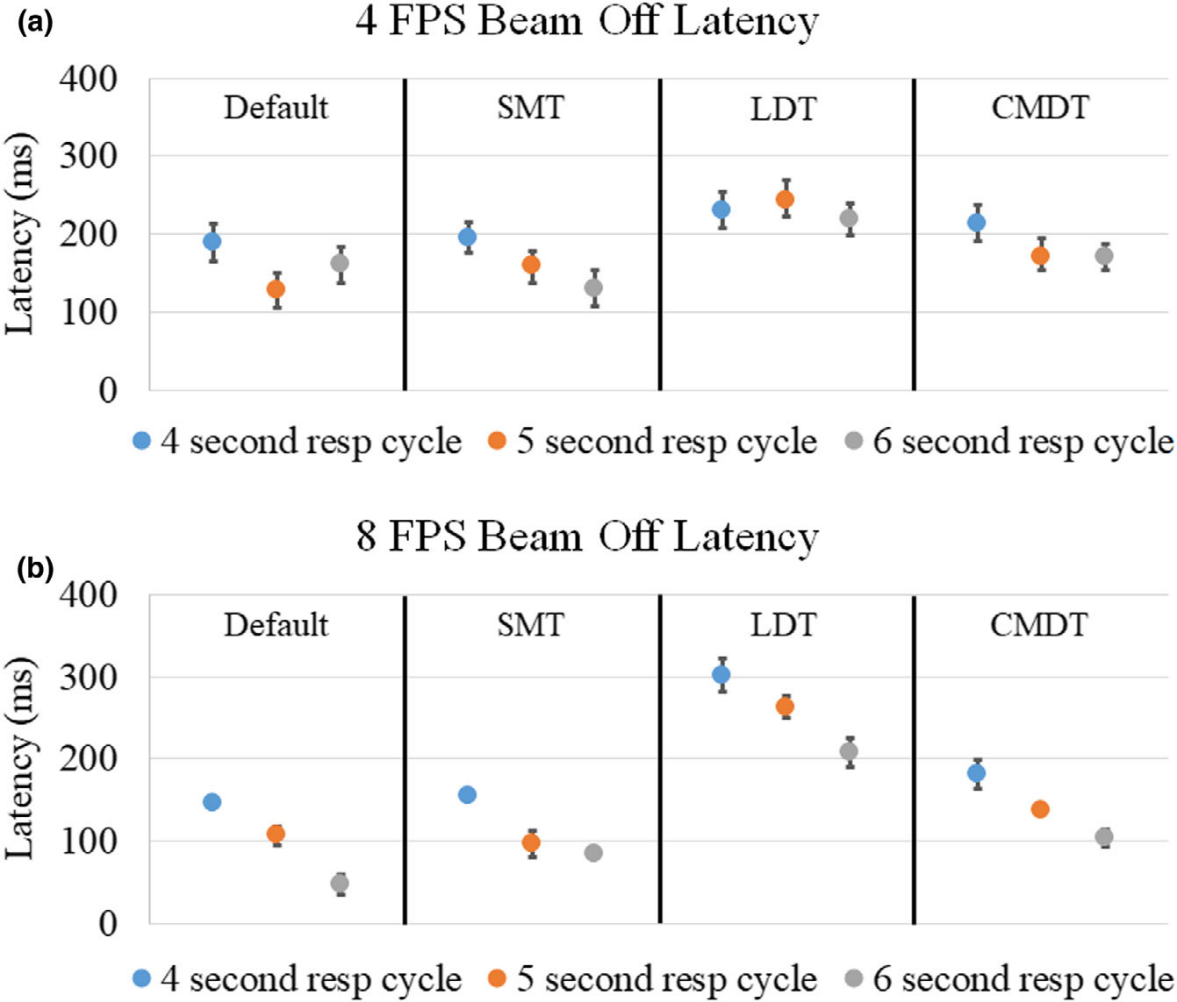
Average (±1 standard error) beam‐off latency values for (a) 4 FPS and (b) 8 FPS acquisition speeds. Vertical black bars separate tracking algorithms for default, small mobile targets (SMT), large deforming targets (LDT), and complex mobile and deforming targets (CMDT).

Average (±standard error) beam‐off latency for the four tracking algorithms on three motion periods are shown in Table 2. The largest latency for 4 FPS imaging was 243 ± 26 ms with the LDT algorithm and a 5 s motion period, and 302 ± 20 ms for 8 FPS imaging with the LDT algorithm and a 4 s motion period. The range of the average beam‐off latency was 128–243 ms in 4 FPS imaging and 47–302 ms in 8 FPS imaging. Figure 3 shows the average beam‐off latency values for all 24 combinations of tracking algorithm, imaging FPS and motion periods. Table 3 shows the results of a two‐sided student’s t‐test of beam‐off latency distributions.

**Table 2:** Average (±1 standard error) beam‐off latency with tracking algorithms on three period motions.

Beam‐off latency (ms)	4 FPS				8 FPS			
	4s	5s	6s	Mean (ms)	4s	5s	6s	Mean (ms)
Default	189 ± 25	128 ± 23	161 ± 23	159	147 ± 2	107 ± 12	47 ± 12	100
Small mobile targets (SMT)	195 ± 20	160 ± 19	130 ± 23	162	154 ± 2	97 ± 16	84 ± 4	112
Large deforming targets (LDT)	231 ± 23	243 ± 26	219 ± 21	231	302 ± 20	263 ± 13	208 ± 18	258
Complex mobile and deforming targets (CMDT)	214 ± 23	171 ± 23	170 ± 17	185	182 ± 18	138 ± 3	104 ± 10	141
Mean (ms)	207	176	170		196	151	111	

**Table 3:** *P*‐values from two‐sided student’s t‐test of beam‐off latency distributions. Statistically significant values (*P* < 0.05) are bolded with gray background, values <0.01 are indicated by <0.01.

Algorithm	Respiratory period	Default		Small mobile targets			Large deforming targets			Complex mobile and deforming targets		
		5	6	4	5	6	4	5	6	4	5	6
4 FPS beam‐off latency *P*‐values												
Default	4	0.96	0.80	0.42	0.83	0.95	0.12	0.07	0.18	0.23	0.70	0.74
	5	–	0.16	**0.02**	0.14	0.48	**<0.01**	**<0.01**	**<0.01**	**0.01**	0.10	0.07
	6	–	–	0.13	0.51	0.82	**0.02**	**0.02**	**0.04**	0.06	0.37	0.37
Small mobile targets	4	–	–	–	0.90	0.98	0.13	0.08	0.21	0.27	0.78	0.82
	5	–	–	–	–	0.84	**0.01**	**0.01**	**0.02**	**0.04**	0.36	0.35
	6	–	–	–	–	–	**<0.01**	**<0.01**	**0.01**	**0.01**	0.11	0.08
Large deforming targets	4	–	–	–	–	–	–	0.36	0.64	0.6	0.96	0.98
	5	–	–	–	–	–	–	–	0.76	0.80	0.97	0.99
	6	–	–	–	–	–	–	–	–	0.56	0.93	0.96
Complex mobile and deforming targets	4	–	–	–	–	–	–	–	–	–	0.90	0.93
	5	–	–	–	–	–	–	–	–	–	–	0.51
8 FPS beam‐off latency *P*‐values												
Default	4	0.99	1.00	**0.02**	0.99	1.00	**<0.01**	**<0.01**	**<0.01**	**0.03**	0.99	1.00
	5	–	0.99	**<0.01**	0.69	0.94	**<0.01**	**<0.01**	**<0.01**	**<0.01**	**0.01**	0.57
	6	–	–	**<0.01**	**0.01**	**<0.01**	**<0.01**	**<0.01**	**<0.01**	**<0.01**	**<0.01**	**<0.01**
Small mobile targets	4	–	–	–	0.99	1.00	**<0.01**	**<0.01**	**<0.01**	0.06	1.00	1.00
	5	–	–	–	–	0.75	**<0.01**	**<0.01**	**<0.01**	**<0.01**	**0.01**	0.36
	6	–	–	–	–	–	**<0.01**	**<0.01**	**<0.01**	**<0.01**	**<0.01**	**0.05**
Large deforming targets	4	–	–	–	–	–	–	0.94	0.99	1.00	1.00	1.00
	5	–	–	–	–	–	–	–	0.99	0.99	1.00	1.00
	6	–	–	–	–	–	–	–	–	0.85	1.00	1.00
Complex mobile and deforming targets	4	–	–	–	–	–	–	–	–	–	0.99	0.99
	5	–	–	–	–	–	–	–	–	–	–	0.99

Average (±standard error) beam‐on latency for the four tracking algorithms with three motion periods are shown in Table 4. The largest latency for 4 FPS imaging was 464 ± 23 ms with the Default algorithm and a 4 s motion period and 785 ± 16 ms for 8 FPS imaging with the SMT algorithm and a 5 s motion period. The range of the average beam‐on latency was 342–464 ms with 4 FPS imaging and 664–785 ms with 8 FPS imaging.

**Table 4:** Average (±1 standard error) beam‐on latency with tracking algorithms and three motion periods.

Beam‐on latency (ms)	4 FPS				8 FPS			
	4 s	5 s	6 s	Mean (ms)	4 s	5 s	6 s	Mean (ms)
Default	464 ± 23	342 ± 23	401 ± 21	403	704 ± 2	778 ± 21	764 ± 10	749
Small mobile targets (SMT)	449 ± 20	389 ± 25	380 ± 21	406	733 ± 13	785 ± 16	764 ± 22	761
Large deforming targets (LDT)	394 ± 19	369 ± 20	426 ± 81	396	664 ± 2	729 ± 2	703 ± 18	699
Complex mobile and deforming targets (CMDT)	445 ± 24	408 ± 30	375 ± 18	409	705 ± 10	762 ± 19	745 ± 18	737
Mean (ms)	438	377	396		701	764	744	

### 3.B Gating uncertainty determination

Gating uncertainty based on the target motion speed and average beam‐off latency was determined in Table 5. The largest uncertainty was 2.3 mm for 4 FPS imaging with the LDT algorithm and a 4 s motion period and 3.0 mm for 8 FPS imaging with the LDT algorithm and a 4 s motion period. The range of the uncertainty was 1.0–2.3 mm in 4 FPS imaging and 0.3–3.0 mm in 8 FPS imaging.

**Table 5:** Gating uncertainty based on the target motion speed and average beam‐off latency.

Uncertainty (mm)	4 FPS				8 FPS			
	4 s	5 s	6 s	Mean (mm)	4 s	5 s	6 s	Mean (mm)
Default	1.9	1.0	1.1	1.3	1.5	0.9	0.3	0.9
Small mobile targets (SMT)	2.0	1.3	0.9	1.4	1.5	0.8	0.6	1.0
Large deforming targets (LDT)	2.3	1.9	1.5	1.9	3.0	2.1	1.4	2.2
Complex mobile and deforming targets (CMDT)	2.1	1.4	1.1	1.5	1.8	1.1	0.7	1.2
Mean (mm)	2.1	1.4	1.1		2.0	1.2	0.7	

## 4 DISCUSSION

In our institution, the gating latency measurements of a 0.35‐T MR‐LINAC were completed using an MRI^4D^ QUASAR motion phantom after the MRgRT system was upgraded with four tracking algorithms and two imaging speeds.

The average beam‐off latency with the Default algorithm in 4 FPS cine MRI was 189 ± 25 ms for a 4‐s motion period, 128 ± 23 ms for a 5‐s motion period and 161 ± 23 ms for a 6‐s motion period using the MRI^4D^ QUASAR motion phantom. In the previous study, Green et al. reported the average latency of 394 ms (246–527 ms) for 4 s motion in 4‐FPS cine MRI using a CIRS motion phantom in a 0.35‐T MRgRT system (MR‐^60^Co). They utilized a physical sensor to detect motion of the imaging insert and generate the gating signal sent to the oscilloscope while the synchronized gating signal from the MRgRT system image processing routine was sent to the beam delivery system. Once the beam‐off signal from the MRgRT system was sent to the oscilloscope, the screen of the oscilloscope was captured to measure the beam‐off latency. In contrast, our study utilized a single software system to control motion of the imaging insert which generated the gating signal recorded by the software without an oscilloscope. The same synchronized gating signal produced by the MRgRT system image processing routine was sent to the beam delivery system and the beam‐control signal from the MRgRT system was recorded by the software. In this study, the beam‐control latency was measured using a single software system compared to the previous study which required the control software, physical sensor, and oscilloscope.

**Fig. 4:**
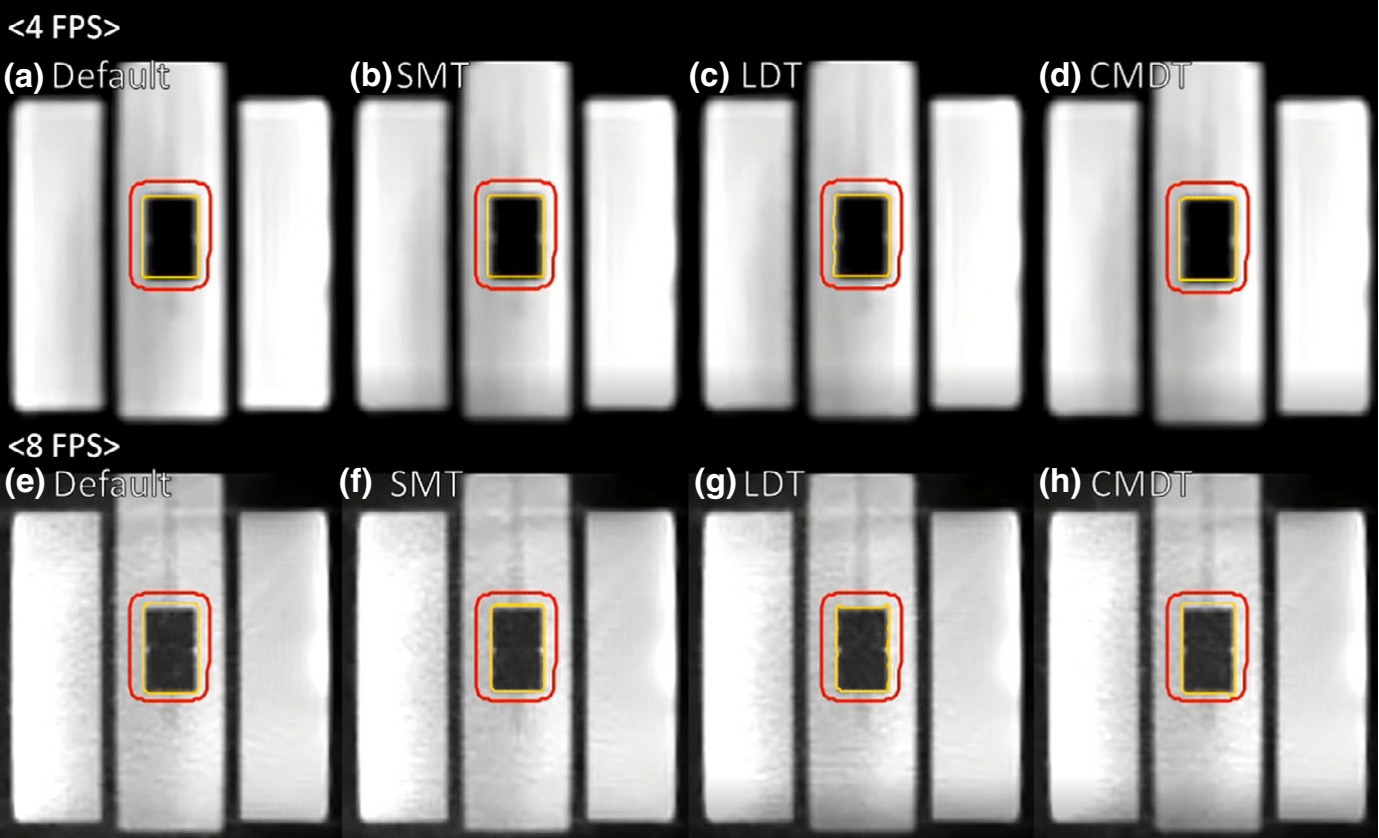
Cine MRI frames from 4 FPS (a–d) and 8 FPS (e–h) acquisitions with the default (a and e), small mobile targets (b and f), large deforming targets (c and g), and complex mobile and deforming targets (d and h) tracking algorithms.

In this study, we measured the beam‐control latency under 24 different scenarios: three phantom motion periods, two imaging speeds and four tracking algorithms, since the gating latency is affected by speed of image acquisition and processing, accuracy and efficacy of target tracking algorithms, and radiation beam delivery latency. First, the imaging time can have an intrinsic latency because images were displayed and processed discretely. The time interval depended on the imaging acquisition and processing. For example, the beam‐off latency was 159 ms in 4 FPS cine MRI but was reduced to 100 ms in 8 FPS cine MRI with Default tracking algorithm. Utilizing, however, view‐sharing for 8 FPS cine MRI did not always improve the gating latency, demonstrating that a high frame rate does not directly translate to improved latency. Second, detection of the target in the image processing routine of the MRgRT system is dependent on the accuracy and efficacy of target tracking algorithms. Since the target contour was generated using target tracking algorithms in real‐time, uncertainty of the target contour delayed the beam‐control decision. For example, the beam‐off latency was 231 ms using the Large Deforming Targets (LDT) tracking algorithm and was reduced to a 159‐ms latency when using the Default tracking algorithm in 4 FPS cine MRI. The LDT tracking algorithm was developed for large targets with a small motion but a large deformation such as whole organs (prostate, bladder, liver, and lung), so the test imaging insert may not be appropriate to utilize the algorithm. Third, the phantom motion speed can affect the distance traveled by the target between each cine MRI acquisition during the gating process. Figure 4 shows a cine MRI frame from 4 FPS and 8 FPS and all four algorithms with the gating target at the end of exhalation position. Yellow contours show the gating contour, which does not always match the target, such as in Figs. 4(e) and 4(h). For example, with a 20‐mm peak‐to‐trough motion, a 250‐ms acquisition (4 FPS) with a 4‐s motion period would result in 2.5 mm of travel between frames and 1.67 mm with a 6‐s motion period. The increased motion speed impacts image quality, such as motion blurring, which may impact the tracking algorithm performance. The beam‐off latency was 207 ms with a 4‐s motion period and reduced to 170 ms with a 6‐s motion period in 4 FPS cine MRI. Imaging quality, image segmentation technique, and vendor specific image postprocessing can all impact the beam off latency, however all beam‐off values in this study were below 500 ms.

The beam‐off gating uncertainty were determined using the target motion speed multiplied by the beam‐off latency. The largest latency occurred with the LDT tracking algorithm when using 8 FPS cine MRI. Gating uncertainty based on the target motion speed was determined to be about 3.0 mm. In our institutional procedure, a target boundary with 3 mm expansion was practical to compensate for the largest beam‐off latency. These calculations are based on the uniform 4 s motion period of the phantom, which is different from real‐world respiratory motion. Respiratory motion follows a variable velocity and has inconsistent amplitude across respiratory cycles. These factors were not considered by this work to isolate the gating latency inherent to the system from additional uncertainty that may be imparted due to variability in motion pattern. However, because the gating position is set to end of exhalation where respiratory position is most consistent and rapid motion is not expected, the experimental setup resembles normal MR‐guided gating conditions. Sampling frequency can impact gating latency, true acquisition speed for 4 FPS images was approximately 210 ms (100 phase encoding lines and TR = 2.1 ms), and the 8 FPS acquisition utilizes view‐sharing so the full k‐space is not acquired within 125 ms. For these reasons it is unlikely that motion phase was matched across all measurements. Further imaging frequencies were not investigated because there are no additional cine acquisition sequences available in clinical mode.

The beam‐on gating latency was much larger than the beam‐off latency measured in this study. This is not as critical clinically due to beam‐on occurring as the target moves fully into the gating boundary, while beam‐off occurs as the target leaves the gating boundary. Greater beam‐on gating latency for 8 FPS, compared to 4 FPS, may be due to the use of view sharing for 8 FPS acquisitions. The view sharing method utilizes previously acquired k‐space lines that may have been acquired while the gating target was further from the gating boundary, thus blurring the edges of the gating target. This blurring would be greater as the gating target enters the boundary because the object has moved further prior to entering the boundary than it would have prior to leaving the boundary. Gating parameter selection also had a more significant impact on the beam‐on latency than beam‐off latency, especially for 4 FPS vs 8 FPS acquisition with the beam‐on latency nearly doubling with 8 FPS acquisition compared to 4 FPS. Gating latency is also dependent on tracking algorithm, which may be due to the efficiency and accuracy of the automatic segmentation and tracking algorithm. A high beam‐on latency time also reduces the treatment duty cycle, potentially increasing treatment time. Reducing beam‐on latency is important for patients who cannot remain in the MRI bore for extended periods of time and for clinic efficiency.

The beam‐control measurements using the MRI^4D^ QUASAR motion phantom was a straightforward process. It did not require any additional software or devices, such as an oscilloscope or dosimetric devices. Measured data were handled by the control software, and comprehensive analyses and corresponding reports were generated in a user‐friendly format as shown in Fig. 2(d). It is noted, however, that uncertainty can be introduced through the gating setup. For example, the imaging insert was shifted 8 mm above the full exhalation position (8 mm below the peak of the 20 mm phantom motion range), to touch the gating boundary in Figure 2(b). If the target‐out was less than 5%, the imaging insert was further shifted inferior within a half‐step of the image resolution (1.75 mm). A consistent target‐out value was used to reduce user‐induced error. Green et al. have previously reported that an ROI value of 5–10% was able to account for tracking contour mismatch.^6^ First, the target‐out rate was not a static number since the quality of cine MRI changed and the corresponding target contour changed. Second, the additional shift was determined by an operator which can reduce the beam‐off latency. In a simple scenario, a 1.75 mm additional shift with 4 s period motion can reduce the beam‐off latency by 175 ms.

As motion gating becomes more prevalent in radiotherapy, continued development of readily deployable QA methods for measuring gating latency, and the associated margins required for treatment, is required. The report from AAPM TG‐76 provides information relevant to current respiratory gating QA and compensating for gating latency. AAPM TG‐76 recommends that the total time delay for real‐tie tracking or compensation systems should be no more than 500 ms because of irregularities in the breathing cycle making tumor position prediction difficult.^16^ Real‐time 2D target tracking without a surrogate remains a rapidly evolving area of study, especially as MRgRT systems become more common. Future methods for gating QA may include the addition of patient‐specific gating latency to determine patient‐specific margins on table. This work provides a new methodology for gating latency measurement which can be applied to newly introduced target tracking algorithms and cine MRI acquisition methods.

## 5 CONCLUSIONS

The gating latency was measured using a MRI^4D^ QUASAR motion phantom on a 0.35‐T MR‐LINAC. The latency measurements include time delay related to MR imaging method, target tracking algorithm and system delay. The gating uncertainty was estimated based on the beam‐off latency measurements and the target motions. The current clinical margin of 3 mm is sufficient to account for gating latencies for all 24 combinations of testing parameters used in this study. Our clinical experience using the MRI^4D^ QUASAR motion phantom on a 0.35‐T MR‐LINAC can be applied to new tracking algorithms, cine MRI acquisition methods, and MRI systems. Further work is required to investigate the impact of irregular motion patterns on gating latency.
